# COMPARISON OF FASCIAL THICKNESS IN HEMIPLEGIC AND NON-HEMIPLEGIC UPPER EXTREMITIES WITH ULTRASONOGRAPHIC IMAGING: A CROSS-SECTIONAL STUDY

**DOI:** 10.2340/jrm.v58.45312

**Published:** 2026-10-08

**Authors:** Nazlı HÖBEK BAŞER, Betül AYDIN, Evrim KARADAĞ SAYGI, İlker YAĞCI, İpek MIDI, Özge KENIŞ COŞKUN

**Affiliations:** 1 Department of Physical Medicine and Rehabilitation, Marmara University Faculty of Medicine, Istanbul; 2 Department of Neurology, Marmara University Faculty of Medicine, Istanbul, Turkey

Hemiplegia following stroke leads to complex neuromuscular and structural changes that impair upper-extremity function. Spasticity is one of the most prominent features and is associated not only with altered neural control but also with secondary changes in muscle and connective tissues. Stroke is one of the leading causes of long-term disability worldwide, particularly among elderly individuals and those with chronic diseases such as hypertension and diabetes. With the ageing of the global population, the incidence of stroke continues to rise (2). In hemiplegic patients, the clinical presentation varies depending on the severity and location of the lesion. Common findings include muscle weakness, spasticity, abnormal muscle synergies, joint contractures, reduced mobility, and limited range of motion (3).

It has been suggested that spasticity may lead to increased fascial thickness, yet current literature provides limited evidence on this subject (4). Fascial thickness in the upper extremity is a non-invasive parameter that can be reliably assessed by ultrasonography, as defined by Pirri et al. (5). Recent studies suggest that spasticity and reduced mobility may contribute to structural remodelling of the fascia, including increased thickness and stiffness due to fibrosis and chronic low-grade inflammation. However, there are no studies demonstrating whether intercompartmental fascial thickness is affected in hemiplegic patients, or its association with spasticity and functional status. Understanding fascial involvement may also have clinical implications, particularly in interventions such as botulinum toxin injections, which aim to reduce spasticity and prevent secondary soft-tissue changes. Therefore, the aim of this study was to evaluate forearm fascial thickness ultrasonographically in both the affected and unaffected upper extremities of hemiplegic patients and to identify possible differences.

## MATERIAL AND METHODS

This cross-sectional observational study was conducted in the Department of Physical Medicine and Rehabilitation of a tertiary hospital between 2023 and 2024. Patients aged 18–75 years with hemiplegia secondary to a cerebrovascular event at least 6 months earlier were included. Exclusion criteria included connective tissue or skin diseases, botulinum toxin injection within the past 3 months, and upper-extremity injuries (fracture, tendinopathy, tendon rupture). All participants provided written and verbal informed consent. The study was approved by the local ethics committee and conducted according to the Declaration of Helsinki.

### Clinical assessments

Spasticity was assessed using the Modified Ashworth Scale (MAS), motor recovery was classified according to the Brunnstrom Stages of Motor Recovery, and upper-extremity functional performance was evaluated with the Fugl-Meyer Assessment (FMA).

The Modified Ashworth Scale (6) was used to quantify resistance to passive movement in major joints of the affected upper extremity, including the shoulder, elbow, wrist, and fingers. Each joint was passively moved through its available range of motion by a single experienced physiatrist, and muscle tone was scored on a 6-point ordinal scale (0–4, with 1+ as an intermediate value). A score of 0 indicated normal tone, while 4 indicated a rigid limb in flexion or extension. The total score represented the global severity of spasticity in the upper limb.

Motor recovery was further classified using the Brunnstrom Stages (7), which describe 6 sequential phases of recovery from flaccidity to near-normal movement patterns. Each patient was assigned a stage according to their observed motor control level, providing a qualitative description of recovery alongside quantitative scales.

The Fugl-Meyer Assessment (Upper Extremity Subscale) (8) was administered to evaluate sensorimotor integration, voluntary movement, coordination, and reflex activity. The test consists of 33 items scored from 0 (cannot perform) to 2 (performs fully), with a maximum score of 66 points representing normal motor function. All evaluations were performed by the same trained physiatrist on the same day as the ultrasonographic measurements to ensure consistency.

### Ultrasonographic measurements

All fascial measurements were performed following the standardized protocol defined by Pirri et al. (5). Participants were seated on an examination chair with both shoulders in a neutral position, elbows flexed at 90°, and forearms resting comfortably on a table in a mid-prone position to minimize tension on the surrounding soft tissues.

A high-frequency linear-array ultrasound transducer (7–15 MHz) was used in B-mode with minimal probe pressure to avoid artificial compression of the fascial layers. Ultrasonographic evaluation was performed on both anterior (flexor compartment) and posterior (extensor compartment) regions of the forearm, at 5 cm and 10 cm distal to the lateral epicondyle. The transducer was aligned perpendicular to the skin to ensure consistent image acquisition and optimal visualization of the fascial planes.

At each site, the superficial and deep fascial layers were identified as hyperechoic linear structures separating the subcutaneous tissue from the underlying muscle (superficial fascia) and dividing adjacent muscle compartments (deep fascia). The thickness of each fascia was measured as the perpendicular distance between the inner and outer hyperechoic borders.

Three consecutive measurements were obtained at each site by the same experienced ultrasonographer, and the mean of these readings was used for analysis. To minimize intra-observer variability, all measurements were taken during quiet respiration with the limb fully relaxed (Fig. 1).

**Fig. 1:**
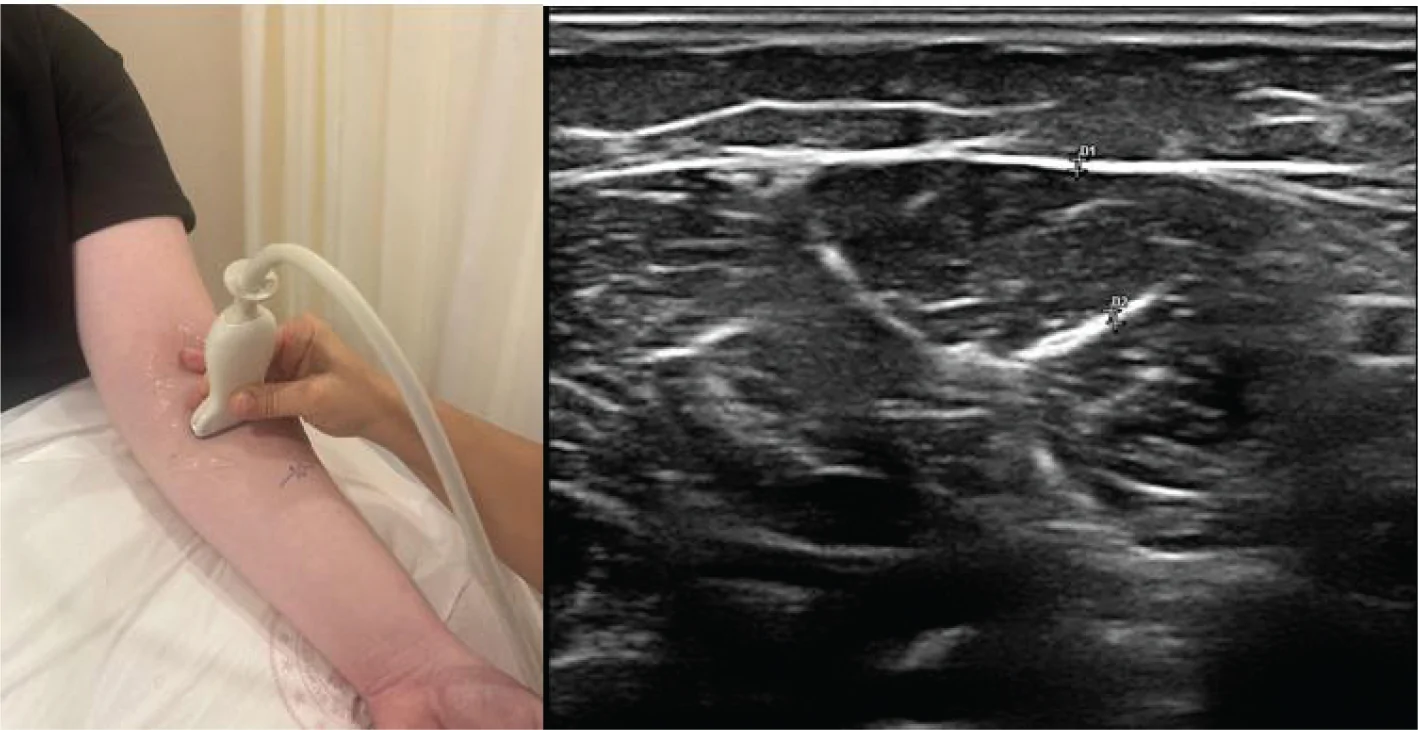
Representative image showing probe placement and an ultrasonographic screenshot.

### Statistical analysis

All statistical analyses were performed using IBM SPSS Statistics v20.0 (IBM Corp, Armonk, NY, USA). Descriptive statistics were used for demographic and clinical data. Normality was tested with the Kolmogorov–Smirnov test. Because the data were normally distributed and each patient was compared with their contralateral side, the paired-samples *t*-test was applied. Measurement reliability was analysed using a 2-way mixed intraclass correlation coefficient (ICC). Correlations between fascial thickness, spasticity, and functional scores were tested with Spearman’s rank correlation. According to Akoglu (9), a correlation coefficient below 0.4 was considered weak, 0.4–0.7 moderate, 0.7–0.8 good, and > 0.8 very good. A *p*-value < 0.05 was considered statistically significant.

## RESULTS

A total of 40 patients were included. The mean (SD) age was 59.9 ± 10.9 years (range 30–75); 10 (25%) were female. Left-sided hemiplegia was observed in 23 patients (57.5%). The mean time from stroke onset was 48.3 ± 35.6 months (range 6–124).

Fascial thickness was significantly greater on the hemiplegic side compared with the contralateral side. Across all measured regions, there was an average difference of approximately 0.10 cm. Mean (SD) values for each measurement site are shown in Table I. The ICCs indicated good intra-rater reliability for both sides (Table II).

**Table I:** Comparison of hemiplegic upper extremity fascia thickness with the unaffected upper extremity. All measurements are in cm

Site	Hemiplegic side fascia thickness Mean (SD)	Unaffected side fascia thickness Mean (SD)	*p*-value (95% CI)
Forearm anterior superficial elbow 5 cm distal	0.69 (0.14)	0.59 (0.11)	*p* = 0.01 (0.04–0.15)
Forearm anterior superficial elbow 10 cm distal	0.67 (0.11)	0.57 (0.10)	*p* < 0.001 (0.05–0.13)
Forearm posterior superficial elbow 5 cm distal	0.67 (0.12)	0.56 (0.12)	*p* < 0.001 (0.06–0.14)
Forearm posterior superficial elbow 10 cm distal	0.65 (0.09)	0.58 (0.12)	*p* = 0.02 (0.02–0.11)
Forearm anterior deep elbow 5 cm distal	0.64 (0.16)	0.50 (0.90)	*p* < 0.001 (0.10–0.20)
Forearm anterior deep elbow 10 cm distal	0.61 (0.09)	0.52 (0.10)	*p* < 0.001 (0.06–0.12)
Forearm posterior deep elbow 5 cm distal	0.60 (0.12)	0.49 (0.10)	*p* < 0.001 (0.07–0.15)
Forearm posterior deep elbow 10 cm distal	0.62 (0.10)	0.51 (0.09)	*p* = 0.001 (0.07–0.14)

SD: standard deviation; CI: confidence interval.

**Table II:** Intraclass correlation coefficients (ICC) of fascia thickness measurements done by the same ultrasonographer on the affected side

Site	Hemiplegic side		Unaffected side	
	ICC	95% CI	ICC	95% CI
Anterior superficial fascia 5 cm distal	0.74	0.60–0.84	0.83	0.74–0.90
Anterior superficial fascia 10 cm distal	0.80	0.69–0.88	0.77	0.65–0.86
Posterior superficial fascia 5 cm distal	0.76	0.49–0.89	0.76	0.64–0.85
Posterior superficial fascia 10 cm distal	0.65	0.49–0.78	0.75	0.62–0.85
Anterior deep fascia 5 cm distal	0.76	0.63–0.85	0.72	0.59–0.83
Posterior deep fascia 5 cm distal	0.88	0.71–0.92	0.76	0.63–0.85
Anterior deep fascia 10 cm distal	0.84	0.75–0.90	0.74	0.61–0.84
Posterior deep fascia 10 cm distal	0.74	0.60–0.84	0.86	0.78–0.91

CI: confidence interval.

Weak but significant correlations were found between: (*i*) elbow-flexor spasticity and anterior superficial fascia measured 10 cm distal to the elbow (ρ = 0.36, *p* = 0.02); (*ii*) finger-flexor spasticity and posterior deep fascia (ρ = 0.32, *p* = 0.04); and (*iii*) pronator spasticity and anterior deep fascia 5 cm distal to the elbow (ρ = 0.36, *p* = 0.02). No other significant correlations were detected between fascial thickness and Fugl-Meyer or spasticity levels (Table III).

**Table III:** Correlation coefficients and *p*-values between fascia thickness and clinical measurements. Significant correlations are shown in bold

Site	Elbow flexor spasticity	Finger flexor spasticity	Wrist flexor spasticity	Pronator spasticity	Fugl-Meyer 1	Fugl-Meyer 2	Fugl-Meyer	Fugl-Meyer 4	Fugl-Meyer 5
Anterior superficial fascia 5 cm distal	rho = 0.21 *p* = 0.18	rho = 0.14 *p* = 0.38	rho = 0.24 *p* = 0.12	rho = 0.12 *p* = 0.44	rho = 0.05 *p* = 0.73	rho = 0.07 *p* = 0.65	rho = 0.13 *p* = 0.40	rho = 0.11 *p* = 0.46	rho = 0.02 *p* = 0.90
Anterior superficial fascia 10 cm distal	**rho = 0.36** ***p* = 0.02**	rho = 0.18 *p* = 0.28	rho = 0.11 *p* = 0.49	rho = 0.28 *p* = 0.08	rho = 0.28 *p* = 0.07	rho = 0.17 *p* = 0.27	rho = 0.07 *p* = 0.65	rho = 0.12 *p* = 0.45	rho = 0.21 *p* = 0.18
Posterior superficial fascia 5 cm distal	rho = 0.15 *p* = 0.36	rho = 0.08 *p* = 0.63	rho = 0.06 *p* = 0.70	rho = 0.28 *p* = 0.07	rho = 0.2 *p* = 0.86	rho = 0.13 *p* = 0.42	rho = 0.03 *p* = 0.98	rho = 0.61 *p* = 0.70	rho = 0.09 *p* = 0.56
Posterior superficial fascia 10 cm distal	rho = 0.23 *p* = 0.16	rho = 0.23 *p* = 0.14	rho = 0.12 *p* = 0.45	rho = 0.18 *p* = 0.26	rho = 0.11 *p* = 0.46	rho = 0.08 *p* = 0.82	rho = 0.05 *p* = 0.71	rho = 0.15 *p* = 0.33	rho = 0.01 *p* = 0.99
Anterior deep fascia 5 cm distal	rho = 0.11 *p* = 0.47	rho = 0.03 *p* = 0.56	rho = 0.27 *p* = 0.08	**rho = 0.36** ***p* = 0.02**	rho = 0.33 *p* = 0.40	rho = 0.32 *p* = 0.82	rho = 0.14 *p* = 0.31	rho = 0.21 *p* = 0.18	rho = 0.12 *p* = 0.45
Posterior deep fascia 5 cm distal	rho = 0.29 *p* = 0.07	rho = 0.03 *p* = 0.82	rho = 0.01 *p* = 0.95	rho = 0.06 *p* = 0.67	rho = 0.57 *p* = 0.72	rho = 0.02 *p* = 0.88	rho = 0.02 *p* = 0.88	rho = 0.11 *p* = 0.48	rho = 0.16 *p* = 0.30
Anterior deep fascia 10 cm distal	rho = 0.11 *p* = 0.47	rho = 0.08 *p* = 0.61	rho = 0.07 *p* = 0.66	rho = 0.14 *p* = 0.37	rho = 0.29 *p* = 0.85	rho = 0.09 *p* = 0.14	rho = 0.02 *p* = 0.82	rho = 0.08 *p* = 0.61	rho = 0.38 *p* = 0.81
Posterior deep fascia 10 cm distal	rho = 0.25 *p* = 0.11	**rho = 0.32** ***p* = 0.04**	rho = 0.14 *p* = 0.36	rho = 0.30 *p* = 0.05	rho = 0.57 *p* = 0.82	rho = 0.03 *p* = 0.98	rho = 0.36 *p* = 0.82	rho = 0.15 *p* = 0.48	rho = 0.07 *p* = 0.62

Values shown in bold indicate statistically significant correlations (*p* < 0.05).

## DISCUSSION

Ultrasonography has been shown to be a reliable and reproducible method for assessing soft tissue structures, including muscle architecture and fascia. Previous studies have demonstrated high intra- and inter-observer reliability in measuring fascial thickness, supporting the methodological robustness of our approach. This reinforces the validity of our findings and highlights ultrasonography as a practical tool for clinical and research settings.

This study demonstrated that fascial thickness in hemiplegic upper extremities was significantly greater compared with the contralateral side. Moreover, fascial thickening was associated with higher spasticity and reduced motor function as measured by the MAS and FMA. These results suggest that fascial remodelling is an important component of musculoskeletal adaptation after stroke.

Previous studies have examined the structural and functional consequences of hemiplegia, including muscle atrophy, fibre-type changes, and connective-tissue remodelling (10, 11). However, literature focusing specifically on fascia is limited. Most prior research in stroke evaluated muscle parameters – such as pennation angle, thickness, and echogenicity – rather than fascial properties (12, 13).

Our findings align with studies showing that immobilization and spasticity lead to fibrotic changes and stiffness in connective tissue (14, 15). The observed correlations between fascial thickening and spasticity support the hypothesis that increased tone contributes to adaptive fascial remodelling. Clinically, fascial thickening may exacerbate joint range-of-motion limitations and further impair upper-extremity function by reducing muscle compliance. Consequently, rehabilitation strategies targeting muscle tone may benefit from the addition of fascia-oriented interventions such as manual therapy, stretching, or myofascial-release techniques.

Previous research has also explored changes in fascia in other musculoskeletal conditions (16–18), but those pathologies primarily involve local soft-tissue or mechanical abnormalities rather than upper-motor-neuron syndromes. Since spasticity involves both neurophysiological alterations and secondary structural adaptations, the nature of fascial involvement remains an important and largely unexplored topic. Our findings contribute preliminary insight, though further studies with larger cohorts and advanced imaging are needed to confirm these observations.

These findings highlight the potential value of integrating fascial assessment into comprehensive post-stroke rehabilitation, as fascial stiffness may represent a modifiable contributor to upper limb dysfunction.

In conclusion, this pilot study demonstrates that forearm fascial thickness is increased in hemiplegic patients and can be reliably assessed using ultrasonography. These findings suggest that fascial remodelling may play a role in post-stroke musculoskeletal adaptations and may contribute to functional impairment. Incorporating fascial assessment into rehabilitation practice may improve treatment planning. Further longitudinal studies are needed to clarify causal relationships and therapeutic implications.
